# Progesterone alleviates hypoxic-ischemic brain injury via the Akt/GSK-3β signaling pathway

**DOI:** 10.3892/etm.2014.1858

**Published:** 2014-07-21

**Authors:** XIAOJUAN LI, JUNHE ZHANG, SHUJIE CHAI, XIAOYIN WANG

**Affiliations:** 1 Department of Physiology and Neurobiology, Xinxiang Medical University, Xinxiang, Henan 453003, P.R. China; 2 Department of Biochemistry and Molecular Biology, Xinxiang Medical University, Xinxiang, Henan 453003, P.R. China; 3 The Third Affiliated Hospital of Xinxiang Medical University, Xinxiang, Henan 453003, P.R. China

**Keywords:** progesterone, hypoxia ischemia, brain damage, Akt, glycogen synthase kinase 3β

## Abstract

This aim of this study was to investigate whether progesterone (PROG) alleviates the neuronal apoptosis in neonatal rats with hypoxic-ischemic (HI) brain damage through the phosphatidylinositol 3-kinase (PI3K)/Akt/glycogen synthase kinase-3β (GSK-3β) signaling pathway. A total of 96 newborn Wistar rats aged 7 days were randomly divided into four groups: sham surgery, HI, drug prevention (PROG) and Akt inhibitor groups. HI animal models were established by a conventional method. All animals were sacrificed 24 h after hypoxia. Immunohistochemistry was used to detect the distribution and expression of phosphorylated Akt (p-Akt) and the GSK-3β proteins in the brain, and western blot analysis was used to determine the p-Akt and GSK-3β protein contents. An enzyme-linked immunosorbent assay was also used to determine the GSK-3β content of the brain tissue, and flow cytometry was used to evaluate the apoptosis rate of neural cells. The expression of p-Akt protein was reduced in the brain tissues of the HI group, whereas GSK-3β expression was increased. In addition, the GSK-3β content of the brain and the neuronal apoptosis rate were significantly increased. PROG pre-treatment increased p-Akt expression, decreased GSK-3β expression and GSK-3β content, and also reduced neuronal apoptosis. Following administration of the Akt inhibitor wortmannin, p-Akt expression decreased, GSK-3β expression increased, and the GSK-3β content and neuronal apoptosis rate significantly increased (P<0.05). In conclusion, PROG activates the PI3K/Akt/GSK-3β pathway to promote Akt activation, enhance p-Akt expression and inhibit GSK-3β expression, thereby inhibiting neuronal apoptosis, alleviating HI brain injury and inducing a cerebroprotective effect.

## Introduction

The inflammatory response induced by hypoxic-ischemic (HI) brain damage may further trigger the activation of certain signaling pathways, finally leading to cell apoptosis or necrosis (1,2). Various complex chemical neurotransmitters and signaling pathways are involved in this process. A large number of studies have confirmed that the phosphatidylinositol 3-kinase (PI3K)-protein kinase B (Akt) signaling pathway is one of the major pathways involved in neuronal apoptosis in the brain (3,4). The PI3K/Akt signaling pathway is an important mode of transducing cell membrane receptor signals into intracellular signals, and thus serves a key function in maintaining cell survival and inhibiting apoptosis. This pathway is also involved in regulating cell proliferation and inhibiting apoptosis by affecting the activation of effector molecules, such as cyclin and apoptosis-related proteins downstream (4).

Glycogen synthase kinase-3β (GSK-3β) is an essential protein downstream of Akt that is involved in the pathological process of HI brain damage. The PI3K/Akt signal transduction pathway may affect the activity of GSK-3β. Activated Akt inhibits GSK-3β activity via phosphorylation (5). A study of cerebral ischemic injury has confirmed that GSK-3β serves a pro-apoptotic function in HI brain injury by activating the p53 gene that expresses the P53 protein. P53 protein inhibits anti-apoptotic Bcl-2 and activates pro-apoptotic Bax, thereby promoting apoptosis. GSK-3β may also inhibit heat shock factor 1 (HSF 1) to decrease HSP70 expression and promote apoptosis (6). Therefore, neuronal apoptosis due to HI brain injury in neonatal rats may be alleviated through intervention in the PI3K/Akt/GSK-3β signaling pathway, which may provide new solutions for the treatment of brain injury.

Progesterone (PROG) is generated by endocrine tissues outside the nervous system and acts on reproductive organs to provide a natural regulating effect on reproductive function. However, recent studies suggesting protective effects of PROG on the brain, in spinal cord injury, as well as in peripheral nerve repair after injury, have aroused considerable interest (7–9). A number of studies have found that PROG can reduce brain edema and scavenge free radicals, as well as function as an antioxidant to enhance the cognitive abilities of animals with brain injury (10–12). However, the specific brain protection mechanism of PROG remains unclear, and studies of brain damage in newborn rats are considerably limited. To the best of our knowledge, current literature has not reported on whether PROG alleviates the neuronal apoptosis associated with HI brain damage in neonatal rats by modulating GSK-3β expression via activation of the PI3K/Akt/GSK-3β signaling pathway. Studies investigating this are thus warranted.

In the present study, molecular biology technologies, including immunohistochemistry, western blotting and enzyme-linked immunosorbent assay (ELISA), were used to explore the mechanism by which the PI3K/Akt/GSK-3β pathway induces neuroprotective effects following PROG intervention in HI brain injury. This study also aimed to further clarify the molecular mechanism of the protective effect of PROG and provide experimental and theoretical evidence relevant to the clinical therapeutic applications of PROG.

## Materials and methods

### Animal source and grouping

Ninety-six male and female Wistar rats, aged 7 days and weighing 12–18 g were provided by the Experimental Animal Centre of Xinxiang Medical University (Xinxiang, China). The animals were randomly divided into four groups of 24: Sham surgery, HI, drug prevention (PROG) and Akt inhibitor groups. Neck incision without ischemia and hypoxia treatment was performed on animals in the sham group. Animals in the HI group were subjected to the animal model preparation process described below. Animals in the drug prevention group were subjected to the animal modeling process and injected with 0.5 g/l PROG solution (Sigma-Aldrich, St. Louis, MO, USA) intraperitoneally at a dose of 8 mg/kg 30 min prior to hypoxia. Animals in the Akt inhibitor group were also subjected to the animal modeling process. However, 30 min prior to model establishment, the rats underwent positioning of the left hippocampus using a brain stereotaxic apparatus, and were injected with dilute (0.16 μg/μl) wortmannin solution (Sigma-Aldrich) into the left hippocampus at a dose of 16 μg/kg. This study was carried out in strict accordance with the recommendations in the Guide for the Care and Use of Laboratory Animals of the National Institutes of Health: Eight Edition (2010). The animal use protocol was reviewed and approved by the Institutional Animal Care and Use Committee (IACUC) of Xinxiang Medical University.

### Animal model preparation

Following isoflurane anesthesia, supine neonatal rats were fixed on a rat bench. The skin of the neck was sliced in the middle following disinfection with alcohol, and then the left common carotid artery was isolated from within the sternocleidomastoid muscle, ligated with silk and cut from the middle. The wounds were sutured, and the animals were left to recover at room temperature for 2–3 h. Animals without rotational movement of the left side were removed and placed in a sealed container at a constant temperature of 37°C, and ventilated with 8% O_2_ and 92% N_2_ gas mixture at a rate of 1.5 l/min for 2.5 h to prepare the HI animal models (13). In the sham group, the left common carotid artery was isolated only, and was not subjected to ligation or the hypoxia treatment.

### Immunohistochemistry

Experimental animals were sacrificed 24 h after hypoxia-ischemia, and their brains were immediately taken, segmented from the optic chiasm, placed in 10% formalin and fixed overnight. Following conventional dehydration, the brains were transparently embedded in paraffin and sliced into 5-μm sections. Finally, the slices were dewaxed to water, baked and preserved in a 4°C refrigerator in readiness for p-Akt and GSK-3β immunohistochemical staining.

The streptavidin biotin-peroxidase complex (SABC) immunohistochemical technique was used. p-Akt (Ser 473) and GSK-3β immunohistochemical staining was performed in strict accordance with the instructions of the reagent kit provided by Wuhan Boster Biotechnology Co., Ltd. (Wuhan, China). In the negative control assay, phosphate-buffered saline (PBS) replaced the antibody, and the remaining procedures were conducted as described in the kit’s instructions. The positive expression of p-Akt and GSK-3β proteins was indicated by the cytoplasm and membrane of the cells being stained brownish yellow. Two different fields of the cortex of three animal brain tissue slices were observed under a light microscope (magnification, ×400), and images were randomly captured using a digital camera. An HMIAS-2000 color image analysis system (Tongji Medical College Clear Screen Imaging Inc., Wuhan, China) was used to analyze the staining results and to calculate the mean optical density (MOD).

### Western blot analysis

In total, eight animals from each group were sacrificed 24 h after hypoxia-ischemia; their brains were immediately placed on ice and part of the cortex in the brain-damaged side was obtained for protein detection. After adding cell lysis buffer, the proteins were extracted by centrifugation, with the total tissue proteins being contained in the supernatant. The protein concentration was detected using the bicinchoninic acid (BCA) method. Polyacrylamide gel electrophoresis was performed to isolate the target protein, which was transferred to nitrocellulose. Rabbit anti-rat p-Akt (Ser 473), rabbit anti-rat GSK-3β or anti-β-actin antibodies were added as the primary antibodies after sealing, and the kit used was provided by Beijing Zhongshan Golden Bridge Biotechnology Co., Ltd. (Beijing, China). The secondary antibody (Santa Cruz Biotechnology, Inc., Santa Cruz, CA, USA) was added and the sample was incubated at 4°C overnight. A gel or film transient display system was used to scan the films, and image analysis software (Tocan, Shanghai, China) was used to analyze the optical density value of the target bands. The relative protein content was expressed as the grey ratio of the band representing the target protein to that of the band denoting the internal reference protein.

### Flow cytometry

Flow cytometry was used to determine the apoptosis rate of neural cells. Ischemic brain tissues of eight mice from each group were rapidly stripped on ice. Single-cell suspensions were prepared mechanically, centrifuged at 4°C and 1,200 × g for 10 min, and then the supernatant was discarded. The cells were suspended in 1 ml cold PBS by gentle shaking, centrifuged at 4°C and 1,000 rpm for 10 min, and then the supernatant was discarded. The cells were resuspended in 200 μl binding buffer and 10 μl Annexin V-fluorescein isothiocyanate (FITC) and 5 μl propidium iodide (PI) were then added. The cell suspension was gently mixed and stored in the dark at room temperature for 15 min. Finally, 300 μl binding buffer was added to the suspension, which was analyzed using flow cytometry (FACS 420; BD Biosciences, Franklin Lakes, NJ, USA).

### ELISA

A total of eight animals in each group were sacrificed 24 h after hypoxia-ischemia. Following sacrifice, the craniotomy raphe nucleus, stripped pia mater and ischemic brain tissue were placed in a homogenizer. Following the addition of 2 ml PBS solution to prepare a tissue homogenate, the tissues were centrifuged at 1,000 rpm for 10 min. The supernatant was obtained, the number of cells was adjusted to between 10^5^ and 10^6^, and then the GSK-3β content was measured by ELISA. The kit was provided by Adlitteram Diagnostic Laboratories (San Diego, CA, USA). A Hyperion MR III microplate reader was used (Biotek Instruments Inc., Winooski, VT, USA) and the process was conducted strictly in accordance with the instructions of the manufacturer.

### Statistical analysis

All experimental data were analyzed using SPSS 13.0 (SPSS, Inc., Chicago, IL, USA). Measurement data are expressed as means ± standard deviations and comparisons between different groups were analyzed using one-way analysis of variance (ANOVA). Paired comparisons between two groups were analyzed using a t-test, with P<0.05 considered to indicate a statistically significant difference.

## Results

### Immunohistochemistry

**Figure 1:**
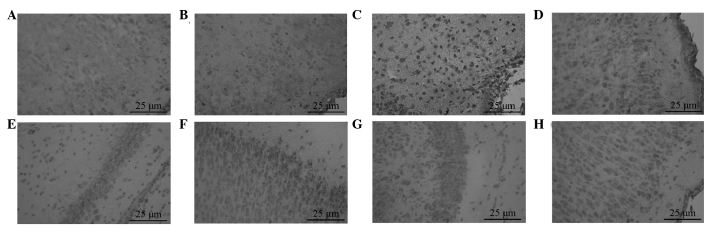
Expression of p-Akt and GSK-3β was observed using immunohistochemistry (magnification, ×400). p-Akt expression in the (A) sham, (B) HI, (C) PROG and (D) Akt inhibitor groups, respectively. GSK-3β expression in the (A) sham, (B) HI, (C) PROG and (D) Akt inhibitor groups, respectively. HI, hypoxic-ischemic; PROG, progesterone; p-Akt, phosphorylated Akt; GSK-3β, glycogen synthase kinase 3β.

Positive staining for p-Akt was revealed as irregularly shaped brown particles, mainly located in the cytoplasm and some in the nucleus. p-Akt-positive cells were occasionally seen in the sham group with light staining, a sparse distribution and low MOD. The expression of p-Akt in the HI group was considerably less, with light staining, a sparse distribution and a lower MOD than that of the sham group; however, the difference between the two groups was not statistically significant (P>0.05). p-Akt expression in the PROG group was the most immunoreactive, with positive particles distributed densely with dark staining and a significantly increased MOD compared with that of the HI group (P<0.05). p-Akt expression in the Akt inhibitor group was significantly decreased compared with that of the PROG group (P<0.05; Table I; Fig. 1A–D).

GSK-3β-positive cells were round- or oval-shaped and were widely distributed in the neurons of the cerebral cortex. GSK-3β expression was infrequently seen in the sham group, and the MOD in this group was low. The expression of GSK-3β in the HI group was significantly higher than that in the sham group (P<0.05). The MOD of the PROG group was significantly lower than that of the HI group, whereas the expression of GSK-3β in the Akt inhibitor group was significantly higher than that in the PROG group (P<0.05; Table I; Fig. 1E–H).

### Western blotting

**Figure 2:**
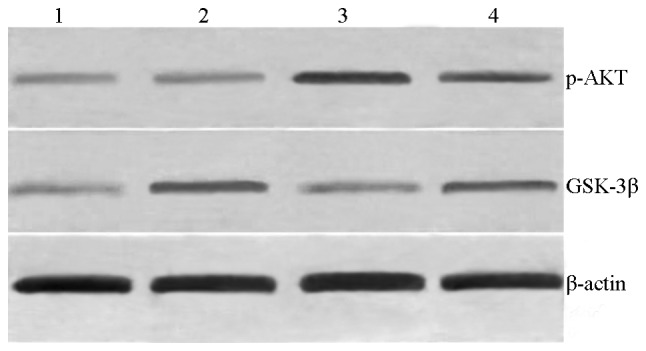
Expression of p-Akt and GSK-3β was observed by western blot analysis. Lane 1, sham group; lane 2, HI group; lane 3, PROG group; lane 4, Akt inhibitor group. GSK-3β, glycogen synthase kinase 3β; HI, hypoxic-ischemic; PROG, progesterone

The western blot analysis results showed that the molecular weight of p-Akt was ~60 kDa. Only a small amount of p-Akt was expressed in the brain tissue of the sham group. The grey ratio of the p-Akt bands with respect to the β-actin internal reference bands in the sham group was insignificantly lower. The p-Akt protein expression level of the PROG group was significantly increased compared with that of the HI group (P<0.05). The level of p-Akt expression in the rat brain tissues was significantly lower compared with that of the PROG group following the use of the Akt inhibitor, and the difference was statistically significant (P<0.05; Table I; Fig. 2). The western blotting results were comparable with the immunohistochemical results for p-Akt in these groups.

The level of GSK-3β expression was low in the sham group, and that of the HI group was significantly higher compared with that of the sham group (P<0.05). The expression level of GSK-3β in the PROG group was significantly lower than that in the HI group (P<0.05). Following the use of the Akt inhibitor, the GSK-3β expression level in the rat brain tissues was significantly increased compared with that of the PROG group, and the difference was statistically significant (P<0.05; Table I; Fig. 2).

### GSK-3β content and neuronal apoptosis rate

The GSK-3β content as determined by ELISA and the neuronal apoptosis rate in the brain tissues of newborn rats in the HI group were significantly higher than those of the sham group (P<0.05). The GSK-3β content and neuronal apoptosis rate in the brain tissue of the PROG-treated group was significantly lower than that of the HI group (P<0.05), whereas the values of in the Akt inhibitor group were significantly higher than those of the PROG group (P<0.05; Table II).

## Discussion

HI brain injury is a critical illness in the neonatal period. Infants are prone to mortality, and survivors easily retain neurological sequelae, which seriously affect their health and quality of life. The pathogenesis of HI brain injury is not fully understood, and no effective treatment is available (14). Therefore, the identification of methods to weaken the effects of cerebral ischemia and protect against neuronal injury is necessary. The pathophysiology of HI brain injury is a complex, multiply linked, multi-factorial, multi-channel cascade of enzymatic activity and injury processes (15), of which nerve cell apoptosis is a major factor. Activation of signal transduction processes is a necessary prerequisite for the initiation of apoptosis.

Studies have suggested that the PI3K/Akt signaling pathway is the main apoptosis-associated signal transduction pathway that is involved following cerebral ischemia, and that this signaling pathway is important in cell survival (4,16). Under physiological conditions, Akt is located in the cytoplasm in a low-activity form; when cells are stimulated by an extracellular signal (for example, ischemia or hypoxia), the *C*-terminal Ser 473 residues of Akt are activated by phosphorylation for the regulation of the upstream molecule. The activated Akt promotes cell survival through a variety of mechanisms, and one of the main mechanisms involves the inhibition of GSK-3β activity to prevent it from exerting an apoptotic effect (17,18). That is, the activated Akt binds to GSK-3β, induces the translocation of GSK-3β to the cell membrane, phosphorylates the *N*-terminal Ser active site, and thereby inactivates GSK-3β (19,20). Therefore, the identification of Akt and GSK-3β as targets for the inhibition of neuronal apoptosis is of great significance for reducing brain damage via the use of neuroprotective agents.

PROG contributes to the protection of brain tissue in the pathogenesis of HI brain injury, and the authors’ previous studies have shown that PROG may antagonize the production of free radicals, inhibit apoptosis, among other activities (9,21). However, the molecular mechanisms and pathways by which PROG inhibits apoptosis remain unclear. Whether the protective effect of PROG against the apoptosis of damaged neurons in the HI brain is realized by modulation of the PI3K/Akt/GSK-3β pathway was worthy of study.

Different degrees of ischemia and various ischemia models generated via the Akt/GSK-3β pathway following cerebral ischemia show different disease states (22,23). Previous studies (23,24) found that p-Akt expression in the neurons of rat models following middle cerebral artery occlusion increased in the 1–3 h after ischemia and reperfusion, with the peak occurring at 1 h, indicating that the PI3K/Akt signaling pathway is involved in the brain ischemia-induced stress response in early ischemia-reperfusion. In transient global cerebral ischemia and focal cerebral ischemia models, the level of p-Akt increased to help to protect neurons against fatal ischemic damage. Following use of a PI3K/Akt inhibitor, ischemic injury worsened. GSK-3β is activated to promote apoptosis in 90-min focal cerebral ischemia models (severe ischemia). Conversely, GSK-3β is inactivated to promote cell survival in short 5-min global ischemia models (mild cerebral ischemia models). Since previous studies (24,25) have shown that the first 24 h for newborn rats with hypoxic brain damage is the most critical, in which the peak time of neuronal apoptosis and the most serious stage of aggravated secondary brain injury occurs, 24 h HI following brain injury was selected as the observation time point in the present study. Changes in the PI3K/Akt/GSK-3β pathway at 24 h following HI brain damage in the neonatal rat brain tissue were observed, and a PI3K/Akt pathway inhibitor, wortmannin, was used to hinder HI brain damage in neonatal rats (wortmannin is a phosphatase inhibitor that prevents Akt phosphorylation and activity) to explore the neuroprotective effect mechanism of the PI3K/Akt/GSK-3β pathway in the process by which PROG attenuates HI brain injury.

To investigate the influence of the Akt/GSK-3β pathway on the HI brain tissue of the neonatal rats, immunohistochemistry and western blotting were used in the present study to detect p-Akt and GSK-3β expression in the brain tissues 24 h following cerebral ischemia, ELISA was used to determine the brain tissue GSK-3β content, and flow cytometry to determine the rate of neuronal apoptosis. p-Akt expression levels were found to be decreased and GSK-3β expression levels were increased in 24 h HI brain tissues. Similarly, the GSK-3β content and neuronal apoptosis rate were elevated, indicating that GSK-3β expression increased to promote apoptosis in the late HI brain period. These results are consistent with those of previous studies (19,20), which suggest that the phosphorylation of Akt decreases following long-term cerebral ischemia, and that GSK-3β is activated to promote apoptosis. Following PROG administration, the highest p-Akt expression level was observed in the PROG group, which exhibited a dense immunoreactive particle distribution, dark staining and significantly increased MOD values compared with those in the HI group. GSK-3β expression levels were significantly reduced, as well as the GSK-3β content of brain tissue, and the neuronal apoptosis rate was lowered, indicating that PROG is able to elevate the levels of p-Akt, inhibit GSK-3β expression and reduce its activity to prevent apoptosis, thus further decreasing HI brain damage. The current study further investigated whether the protective effect of PROG in the brain was achieved through the PI3K/Akt/GSK-3β signaling pathway. Following the intraventricular injection of wortmannin to inhibit the PI3K/Akt pathway, the expression level of p-Akt in the Akt inhibitor group was significantly decreased compared with that of the PROG group, the expression level of GSK-3β was significantly higher than that of the PROG group, and the GSK-3β content and neuronal apoptosis rate were significantly higher than those of the PROG group. These results indicate that the injection of the Akt inhibitor wortmannin aggravates brain damage and confirms that PROG can exert a protective effect during ischemic brain injury through the activation of the PI3K/Akt/GSK-3β pathway, and that the Akt/GSK-3β signaling pathway may mediate the protective effect of PROG on neurons. The protective effect of PROG against brain injury may occur through multiple pathways; therefore, further studies on the underlying associated mechanism are required to provide a theoretical basis for the future prevention and treatment of HI brain disease.

In conclusion, Akt protein activity was decreased in newborn rats in the 24 h following HI brain damage, thereby triggering the activity of its downstream effector GSK-3β, which may be one of the signaling pathways underlying neuronal apoptosis. PROG is able to increase Akt activity, inhibit GSK-3β activity and decrease brain damage. Therefore, PROG is indicated to exert a protective effect on ischemic brain injury by activating the PI3K/Akt/GSK-3β pathway, which provides an experimental basis for its potential use as a drug for treating ischemic brain injury.

## Figures and Tables

**Table I:** Effect of PROG on the expression of p-Akt and GSK-3β in the brain tissue of neonatal rats.

	Immunohistochemistry		Western blotting	
		
Group	p-Akt	GSK-3β	p-Akt	GSK-3β
Sham	0.386±0.022	0.262±0.025	0.21±0.04	0.24±0.03
HI	0.342±0.031	0.738±0.035^a^	0.19±0.06	0.49±0.12^a^
PROG	0.754±0.046^b^	0.432±0.028^b^	0.55±0.16^b^	0.25±0.02^b^
Akt inhibitor	0.429±0.035^c^	0.526±0.021^c^	0.34±0.06^c^	0.40±0.08^c^

Values are presented as the mean ± standard deviation. HI, hypoxic-ischemic; PROG, progesterone; p-Akt, phosphorylated Akt; GSK-3β, glycogen synthase kinase 3β.

a P<0.05 vs. the sham group;

b P<0.05 vs. the HI and sham groups;

c P<0.05 vs. the PROG group.

**Table II:** Effects of PROG on the levels of GSK-3β and rate of apoptosis in brain tissue following HI brain damage in neonatal rats.

Group	GSK-3β (ng/ml)	Apoptosis rate
Sham	3.26±0.21	2.49±0.23
HI	7.87±0.59^a^	10.09±0.36^a^
PROG	5.10±0.42^b^	3.47±0.32^b^
Akt inhibitor	6.65±0.37^c^	6.32±0.56^c^

Values are presented as the mean ± standard deviation. PROG, progesterone; GSK-3β, glycogen synthase kinase 3β; HI, hypoxic-ischemic.

a P<0.05 vs. the sham group;

b P<0.05 vs. the HI and sham groups;

c P<0.05 vs. the PROG group.
